# Weld Zone Analysis Based on FCAW Mechanical Characteristics and Heat Transfer Analysis of 316L Stainless Steel for Liquefied Hydrogen Tanks

**DOI:** 10.3390/ma17112630

**Published:** 2024-05-29

**Authors:** Younghyun Kim, Sungbin Hong, Eulyong Ha, Gyuhae Park, Jaewoong Kim

**Affiliations:** 1 Purpose Built Mobility Group, Korea Institute of Industrial Technology, Gwangju 61012, Republic of Korea; kyh1927@kitech.re.kr (Y.K.); 23hey0302@kitech.re.kr (E.H.); 2 School of Mechanical Engineering, Chonnam National University, Gwangju 61186, Republic of Korea; 3 DT Innovation Planning Department, Hyundai Samho Heavy Industries, Yeongam 58462, Republic of Korea; njjo177@hshi.co.kr

**Keywords:** FCAW (flux cored arc welding), FEA (finite element analysis), heat transfer, 316L

## Abstract

The International Maritime Organization (IMO) is currently rolling out more restrictive regulations in order to achieve net-zero GHG emissions by 2050. In response, the shipping industry is planning to pivot to green energy sources such as hydrogen fuel. However, since hydrogen has an extremely low boiling point (−253 °C), materials for storing liquid hydrogen must be highly resistant to low-temperature brittleness and hydrogen embrittlement. A 316L stainless steel is a typical material that meets these requirements, and various welds have been studied. In this study, 3 pass butt welding was performed by applying the FCAW (flux cored arc welding) process to 10 mm thick ASTM-A240M-316L stainless steel, with the size of the fusion zone and HAZ investigated by mechanical testing and heat transfer FE analysis according to process variables, such as heat input, welding speed, and the number of passes. In all cases, the yield and tensile strengths were about 10% and 3% higher than the base metal, respectively. Furthermore, heat transfer FE analysis showed an average error rate of 1.3% for penetration and 10.5% for width and confirmed the size of the HAZ, which experienced temperatures between 500 °C and 800 °C.

## 1. Introduction

As the effects of global warming increase in intensity, various countries and organizations have been taking steps to reduce greenhouse gas (GHG) emissions. In the maritime transport sector, the International Maritime Organization (IMO) set an initial roadmap in 2018 to reduce GHG emissions by 50% and carbon intensity by 70% relative to 2008. However, the maritime industry has been constantly evolving. As new methods to reduce GHG emissions have been developed, the IMO adopted a revised roadmap in 2023 to reach net-zero emissions by around 2050 [1,2,3].

Additionally, demand has been increasing for green and alternative-fueled vessels in the shipping industry to better transition toward decarbonization [4,5]. Among the alternative energy sources being considered, hydrogen is an ideal option because it does not emit CO_2_ when used as fuel [6,7]. Hydrogen has the advantage of reducing its volume by about 800 times compared to gaseous hydrogen when liquefied, but it has an extremely low boiling point of −253°C [8]. The materials used in liquefied hydrogen tanks for storing liquid hydrogen must have excellent low-temperature brittleness and high resistance to hydrogen embrittlement. Austenitic stainless steel 316L (also known as 316L) is a typical material that meets these requirements. This material is characterized by its high nickel content and low hydrogen solubility due to its FCC structure [9,10,11,12,13,14,15]. However, when exposed to temperatures between 500 °C and 800 °C, microstructural changes can lead to gradual intergranular corrosion. Because of this, research has been conducted on the susceptibility of 316L to intergranular corrosion. Macatangay et al. exposed additively manufactured type 316L to 675 °C for one hour and analyzed the effects on intergranular corrosion susceptibility [16]. Tsai et al. exposed AISI 316L to 700 °C for 10 h and analyzed the precipitation and intergranular corrosion occurring at the grain boundaries [17]. Sahlaoui et al. predicted the susceptibility of AISI 316L to intergranular corrosion depending on the exposure time when exposed to temperatures of 550–700 °C [18].

Welding is an essential process for manufacturing liquefied hydrogen tanks. In this regard, various welding processes have been studied to improve the welding efficiency of 316L. Cho et al. performed heterogeneous butt welding of 20 mm thick 316L and high manganese steel materials with GTAW using different types of filler metals. The resulting welds were compared and analyzed using mechanical testing and microstructure observation at room and cryogenic temperatures [19]. Ha et al. performed BOP welding through fiber laser welding on ASTM-A240M-304, with ASTM-A240M-304 materials under different welding conditions. Additionally, the penetration through cross-sectional analysis of the welds was compared [20]. Tabrizi et al. performed GTAW on 10 mm thick AISI 316L with two modes of continuous and pulsed currents and analyzed the microstructural evolution and phase equilibria of the welded joints [21]. Shreyas et al. performed dissimilar butt welds using GMAW on 2.5 mm thick SAE 316L grade and galvanized steel pertaining to IS277-92 standard material and studied the effects of zinc on the dissimilar welds [22]. Ramakrishnan et al. performed dissimilar butt welding using ND: YAG laser welding on 3 mm thick AISI 316L and Monel 400, a nickel-copper alloy, and obtained the optimal welding conditions through mechanical tests [23]. Yi conducted pipe butt welding on STS 316L and STS Duplex S31803 materials, measured the deformation, and developed a formula to predict deformation based on cross-sectional area and heat input [24]. Park et al. performed laser welding on 5 mm thick carburized 316L material, securing the reliability of the weld through mechanical testing and microstructural analysis [25]. Oh et al. used 316L powder to fabricate layered materials and conducted mechanical analyses based on the fabrication equipment and type of powder [26].

The FCAW (flux cored arc welding) process offers high current density for fast welding speeds and automation, as well as excellent weldability for nickel alloys. Many studies on FCAW studies have been conducted on 316L. Kateherasan et al. performed butt welding on 8 mm thick material and studied the mechanical and metallurgical properties according to changes in the shielding gas ratio [27]. Jang et al. performed butt welding with three types of filler wires on 12 mm thick materials and analyzed the solidification morphology and delta-ferrite content according to the chromium and nickel equivalent ratios (Creq/Nieq) [28]. Li et al. performed butt welding using wet FCAW on 11 mm thick materials with wires containing 24–25 wt.% nickel and analyzed the results through mechanical tests [29]. Kim et al. performed heterogeneous butt welding using FCAW on 16 mm thick 316L and ASTM a516-70 materials under different welding conditions and analyzed them based on mechanical properties and through corrosion tests [30].

Welding processes involve localized heating and cooling of materials, which leads to significant temperature changes and uneven expansion and contraction. This results in welding deformation and stress, which reduces dimensional accuracy and joint strength; negatively affects the reliability of the welded structure; and requires additional work to correct distortions, which can be both costly and time-consuming. Therefore, predicting welding deformation is essential, and studies have been conducted to simulate thermodynamic behavior based on the finite element method (FEM). Urbanski et al. performed butt welding using FCAW on 16 mm thick EH36 steel and compared the welding distortions with 3D FE analysis results [31]. Islam et al. performed 3D FE analysis on the lower arm and lap-joint-shaped welds using 3.2 mm thick ASTM A591 steel and sequentially performed heat transfer and thermo-elastoplastic analysis. Welding conditions that minimize weld-induced distortion were also obtained based on genetic algorithm-based optimization [32]. Pam-nani et al. performed SMAW and GTAW on 10 mm thick DMR-249A steel and measured the weld temperature through mechanical tests and thermocouples. Heat transfer and residual stress analyses were also performed by 3D FE analysis and compared with the experimental results [33]. Deng et al. performed BOP welding using CO_2_ welding on a 2 mm thick Q235 mild carbon steel and studied the welding distortion and buckling characteristics by sequential thermo-elastoplastic analysis [34]. Fu et al. performed fillet welding using MIG welding on 9 mm thick low-carbon-grade DH36 ferric steel and studied the weld deformation and residual stress through sequential thermo-elastoplastic analysis [35]. Pahkamaa et al. performed arc welding on a 17 mm thick austenitic steel plate and compared it with a moving heat source and block dumping technique after thermo-elastoplastic analysis. The block dumping technique is a method that divides the weld into a finite number of blocks and applies heat input magnitude to the blocks one by one in the direction of progress. The results showed high consistency with the results of the moving heat source as the number of block dumps increased [36].

In this study, FCAW welding was performed on 10 mm thick stainless-steel ASTM-A240M-316L under four different welding conditions, and the effects of process variables such as heat input, welding speed, and the number of passes were analyzed through cross-sectional observation and mechanical testing. The results showed that the tensile strength was in the range of about 577 Mpa-611 Mpa, and the impact strength was in the range of about 33–49 J. Afterward, heat transfer FE analysis was performed to predict the fusion zone and HAZ. A 3D FE model was created based on the experimental results, and heat transfer FE analysis was performed by applying the material’s physical properties by temperature. Uniform body heat flux (UBHF) and the block dumping technique were used to simulate the moving heat source. At the beginning of the heat transfer FE analysis, all weld beads were removed, and the heat input magnitude was applied as amplitude over time in the UBHF as blocks were created one by one. As a result of comparing the experimental results of the fusion zone with the heat transfer FE analysis results, the average error rate for penetration was 1.3%, and the average error rate for width was 10.5%. The width of the HAZ that experienced temperatures between 500 and 800 °C was measured, and its size was about 20% larger than that of the fusion zone. The results of predicting the temperature distribution of the weld according to the size of the heat input were somewhat consistent, which demonstrates that using this method can contribute to reducing heat transfer FE analysis time.

## 2. Experimental Conditions

### 2.1. Welding Materials, Equipment, and Conditions

The material used in the experiment was stainless steel ASTM-A240M-316L (POSCO Co., Pohang, Republic of Korea). FCAW welding was performed on two single specimens with a size of 200 × 500 × 10 (width × length × thickness, mm). Table 1 and Table 2 below show the composition, tensile strength, yield strength, and elongation of the base metal used in the experiment.

**Figure 1:**
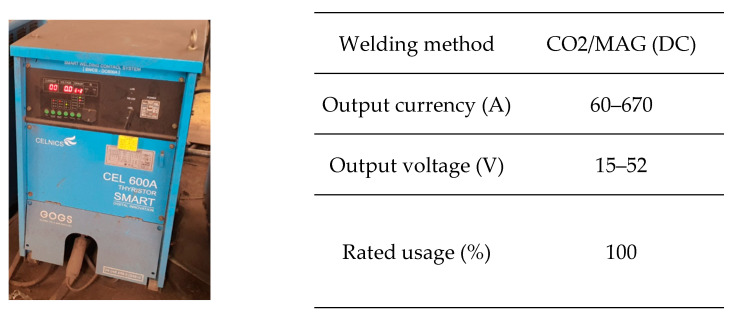
Welding equipment for FCAW.

The CEL 600A (Celnics Co., Geoje, Republic of Korea) CO_2_/MAG welding machine was used in the experiment, and the welding material was SW-316L Cored (1.2 mm, Hyundai Co., Seoul, Republic of Korea). Figure 1 below shows the specifications of the equipment and materials used in the experiment.

**Figure 2:**
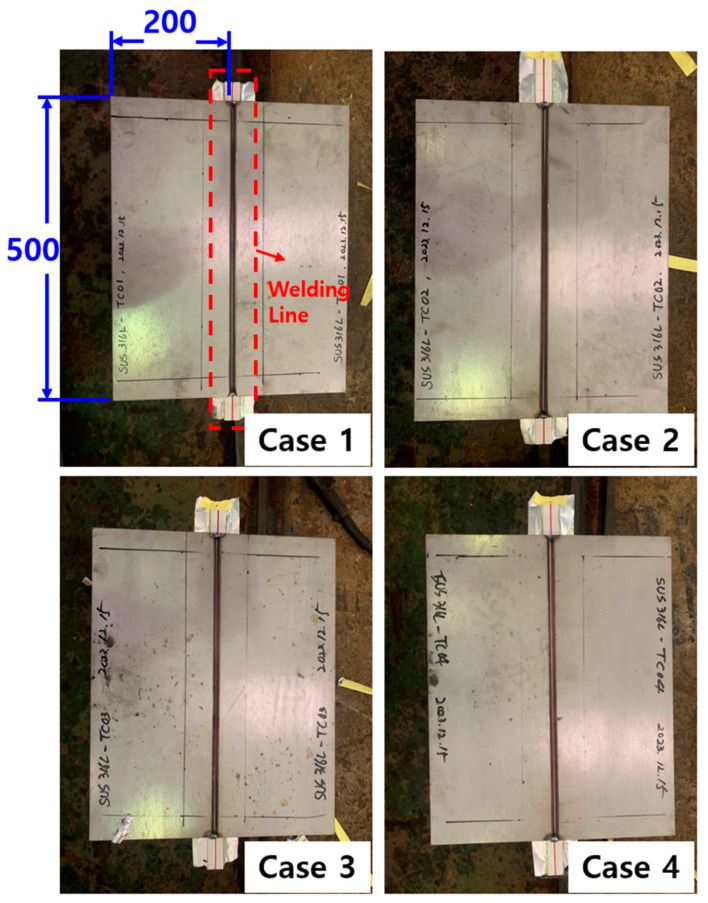
Welding specimen before welding.

**Figure 3:**
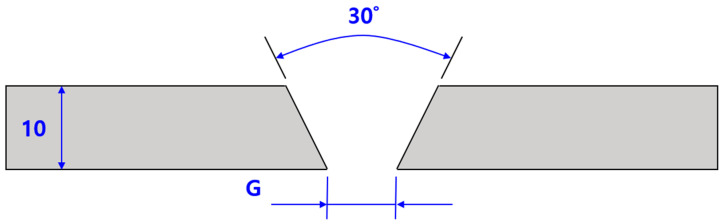
Schematic diagram of welding area cross-section view.

The experiment was performed by making a V-groove configuration at an angle of 30° to the longitudinal plane of two ASTM 316L specimens and then performing FCAW butt welding for a weld length of 500 mm. For butt welding, both ends were tack-welded to minimize step differences between the two specimens during welding, and a ceramic backing material (CERAMIC BACK UP TAPE) was applied to the backside. Figure 2 shows the specimen before welding, and Figure 3 shows a schematic diagram of the welding area.

Four FCAW butt welds were performed on eight single specimens, with the number of welding passes, current, voltage, and speed as variables. The current, voltage, and speed applied in each experiment were selected through tests to secure the optimal butt-welding conditions for the same specimen, and the number of welding passes was divided into one set of two passes and three sets of three passes. Since a set of two passes requires a higher current or voltage and lower welding speed than a set of 3 Passes for proper penetration, this was done to compare the deformation of the material resulting from excessive heat input. Table 3 shows the welding conditions for each experiment.

### 2.2. Microstructural Examination (Deformation Analysis, Cross-Section Observation)

**Figure 4:**
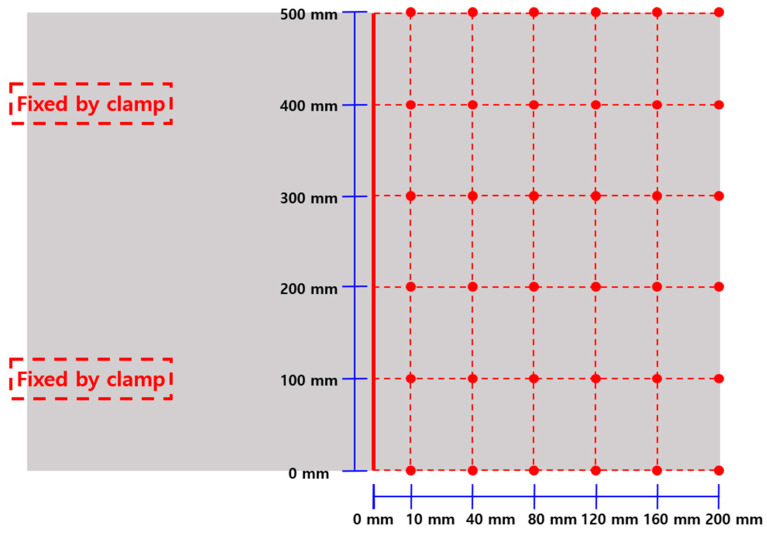
Locations for measuring welding deformation.

**Figure 5:**
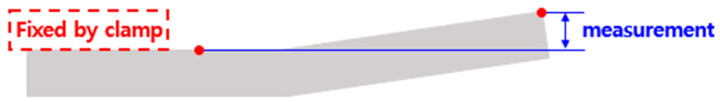
Methods for measuring welding deformation.

As shown in Figure 4 below, the welding deformation of the specimens was measured by clamping the left side of the weld to the floor as a reference point. The upper right side was divided into six sections in both the x- and *y*-axis directions, resulting in a total of 36 measurement points. As shown in Figure 5, the amount of deformation was determined by measuring the height of each measurement point using the floor and the upper surface of the fixed specimen as the reference point.

**Figure 6:**
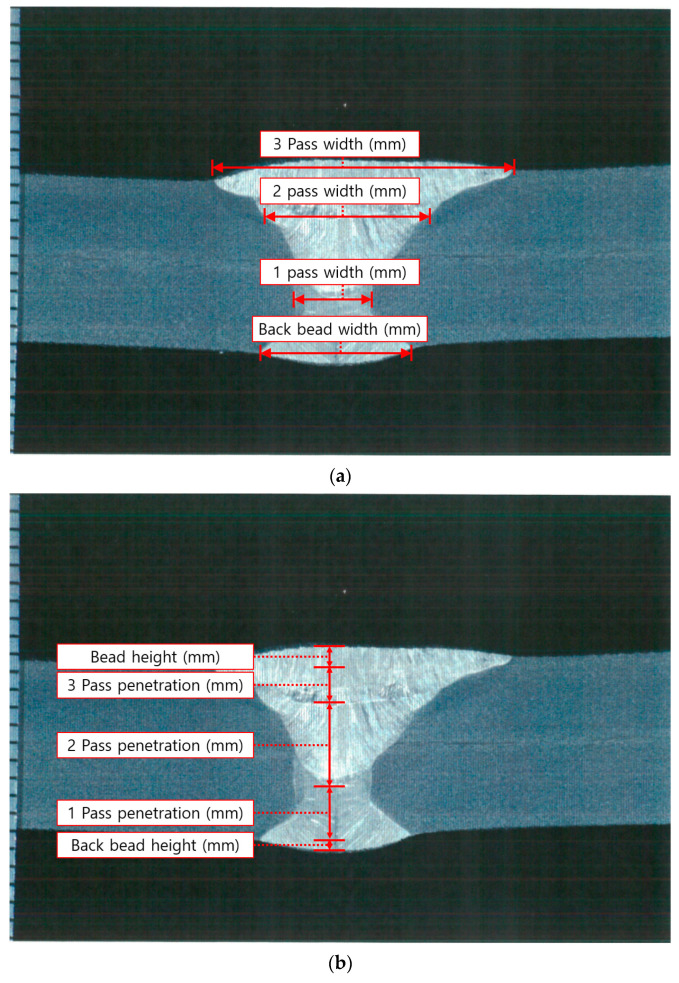
Measurement position of cross-section observation: (**a**) width, (**b**) height and penetration.

**Figure 7:**
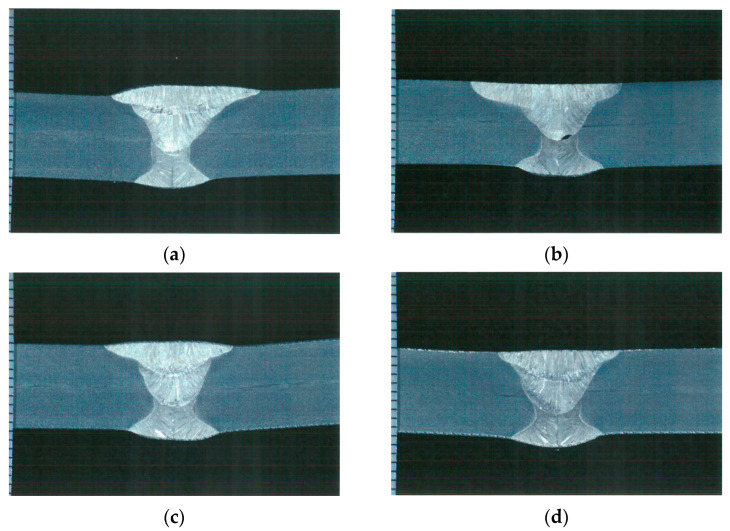
Results of cross-section observation: (**a**) Case 1, (**b**) Case 2, (**c**) Case 3, (**d**) Case 4.

After measuring the welding deformation in each specimen, cross-sectional observation and tensile and impact tests were performed to observe the weld’s bead geometry and examine the mechanical properties. First, the cross-sectional observation was conducted according to ASME Sec. IX—2023 (QW-470). The main parameters (bead width, bead height, and penetration) were measured using an Olympus GX51 microscope, as shown in Figure 6 for each pass. Figure 7 shows the cross-sectional observation results, and Table 4 shows the measurement results for each welding condition.

The tensile and impact tests were conducted in accordance with ASME Sec. II SA370—2023 with a universal tensile tester (DTU-900MHN, maximum 100 kN) and an impact tester (DTU-603B, maximum 600 J) made by Daekyung Tech and Testers Co., Ltd, (Incheon, Republic of Korea).

**Figure 8:**
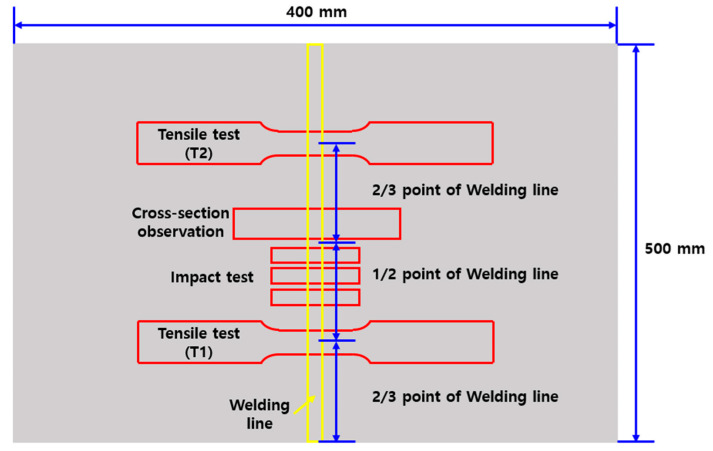
Specimen cutting plan for cross-section observation, tensile test, and impact test.

Two tensile tests and three impact tests were performed on one butt-welded specimen, which was collected as shown in Figure 8 below. The tensile tests were performed at the 1/3 and 2/3 points, and the impact tests were performed on three specimens taken below the 1/2 point.

## 3. Prediction of Welding Deformation Using FEM

A 3D model was created based on the experimental results in Figure 7, and heat transfer FE analysis was performed according to the heat input conditions in Table 3. The temperature distribution in the fusion zone and HAZ was examined through simulation, and the penetration and width size in each case were measured and compared.

### 3.1. Heat Transfer Analysis

The thermal analysis of ABAQUS (Ver. 2020, Dassault Systems Simulia Corp, Johnston, RI, USA) is performed based on the conservation of energy and Fourier’s law, which is shown in Equation (1) below [37,38,39].

(1) $$\int_{V}\rho \frac{\partial U}{\partial t}\delta TdV+\int_{V}\frac{\partial \delta T}{\partial x_{\alpha }}\cdot \left(\lambda \frac{\partial T}{\partial x_{\alpha }}\right)dV=\int_{V}\delta TQdV+\int_{S}\delta Tq_{S}dS$$

Λ = λ(T): Thermal conductivity (W/mK).
∂U/∂t: The material time rate of internal energy (the specific heat, C_p_(T), being given by ∂U/∂T, assuming the volume is held constant).
Q: Volumetric source power (W/m^3^) with laser beam and electric arc volumetric heat sources taken into account.
δT: Variational function.
q_S_: Heat flux toward element surface.
T = T(x_α_,t): Temperature (K).
ρ: Density.

Equation (1) completes the initial conditions t = 0:T = T_0_, Dirichlet, Neumann, and Newton-type boundary conditions and considers heat loss from convection and radiation.

The heat input model used in heat transfer FE analysis was UBHF, the heat input magnitude was applied as amplitude over time, and the moving heat source was simulated using the block dumping technique. The block dumping technique separates the weld into a finite number of blocks in the welding direction and removes all blocks before starting the analysis. Afterward, blocks are created one after another in the welding direction, with heat input magnitudes assigned to the blocks, which requires a shorter analysis time than the moving heat source [36,40].

In this study, the heat input magnitude was applied based on Table 3, and Equation (2) shows the calculation method.

(2) $$Q_{h}=((A\times V\times 60)/W_{s})\times 1/(A_{0})$$

Q_h_: Heat input amount applied per block volume.
A: Current (A).
V: Voltage (V).
W_s_: Welding speed (mm/min).
A_0_: Cross-sectional area by welding pass (mm^2^).

### 3.2. Construction of FEM Model

**Figure 9:**
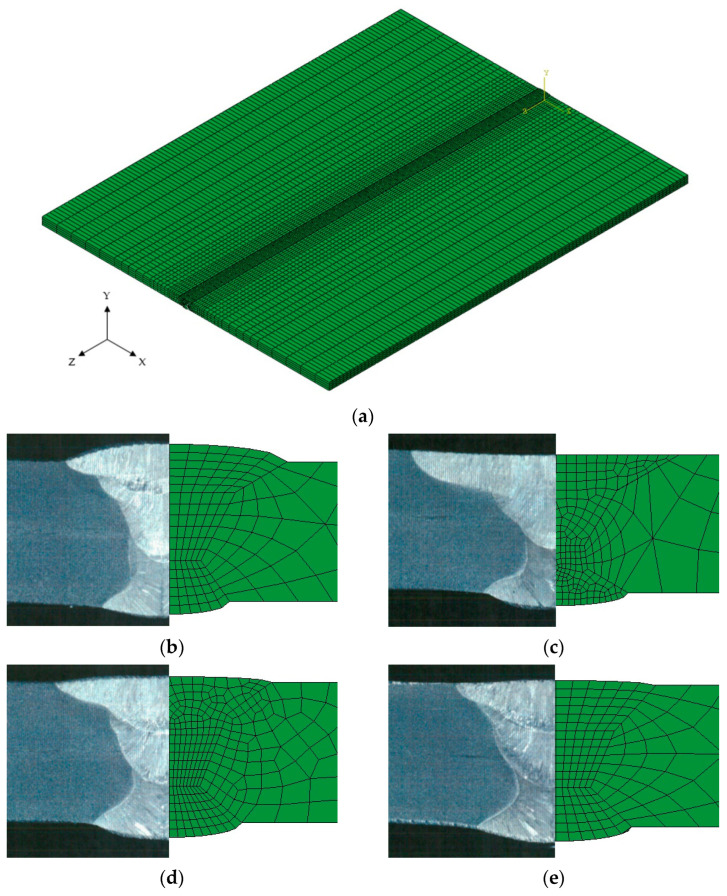
A 3D heat transfer FE model and cross-section: (**a**) 3D finite element model of butt welding using FCAW; (**b**) cross-section of Case 1; (**c**) cross-section of Case 2; (**d**) cross-section of Case 3; (**e**) cross-section of Case 4.

A 3D FE model was constructed based on the cross-sectional observation results analyzed through FCAW butt welding experiments. The dimensions were 400 mm wide, 500 mm long, and 10 mm thick. The average number of elements per case was about 50,000. The minimum size of the element in the weld was 0.7 mm × 5.0 mm × 0.8 mm. The element type used in heat transfer analysis was DC3D8 (an eight-node linear heat transfer brick), and Figure 9 shows the FE model used in the simulation. The ambient temperature was set at 25 °C.

**Figure 10:**
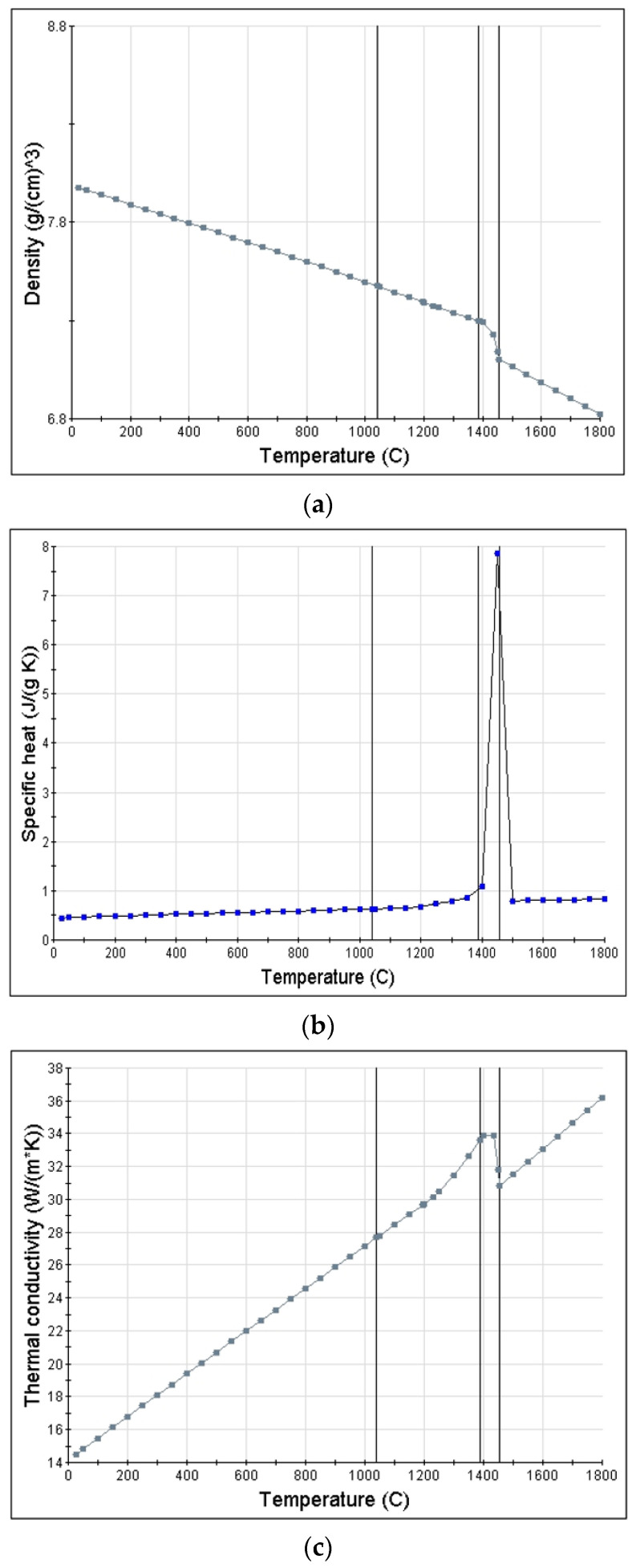
Material properties of ASTM 316L by temperature: (**a**) density, (**b**) specific heat, (**c**) conductivity.

The temperature-specific properties of ASTM 316L were used for the welding heat transfer analysis. The density, specific heat, and thermal conductivity were derived using Jmatpro software, as shown in Figure 10 [41,42].

## 4. Results and Discussion

### 4.1. Results of Tensile, Impact, and Welding Deformation Measurement

Table 5 shows the results of the tensile and yield strength tests on ASTM 316L after applying FCAW welding. As for tensile strength, all four welding conditions showed better strength than the base metal. However, in the case of tensile strength, the value was lower than the base metal at the 1/3 point in Case 2, which corresponds to two passes. In addition, all the fractures that occurred in the weld were ductile failures. In Case 2, the yield strength and tensile strength of the weld zone were lower than the other welding conditions due to the higher current and lower welding speed, resulting in high heat input.

After welding, the specimens were subjected to impact tests in a cryogenic environment (−196 ℃), and Table 6 below shows the results. The impact strength measured was about 34.5–35.6 J in Cases 1, 3, and 4, while in Case 2, the impact strength was 44.7 J. These results were observed to vary depending on the number of welding passes. Condition 2 involved a 2 pass welding process, while Conditions 1, 3, and 4 involved a 3 pass welding process. However, to ascertain precise trends, it is believed that repeated experiments are necessary.

The amount of deformation caused by FCAW welding was measured using the method described in Section 2.2 above, and Table 7 below shows the values. The smallest amount of deformation was observed in Case 2, corresponding to the 2 pass condition, while the highest was in Case 1. Welding distortion is thought to be more significantly influenced by the heat input of the initial welding pass and the number of welding passes, rather than by the total heat input.

### 4.2. Heat Transfer Analysis Results

**Figure 11:**
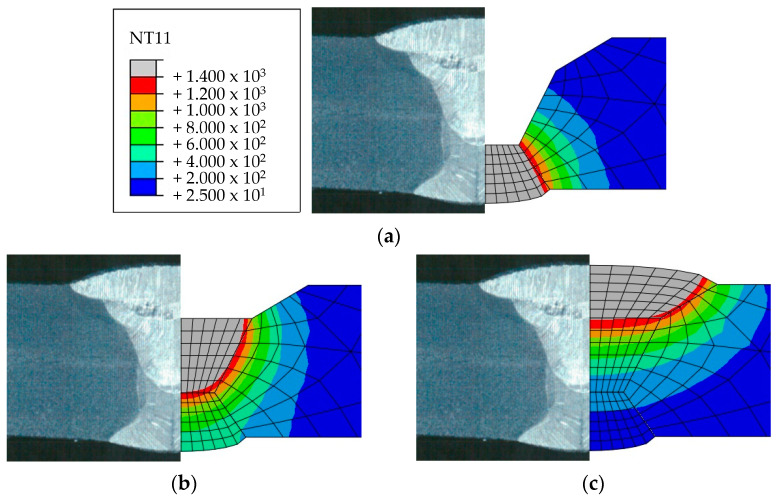
Comparison of experimental results and heat transfer analysis for Case 1: (**a**) 1 pass, (**b**) 2 pass, (**c**) 3 pass.

**Figure 12:**
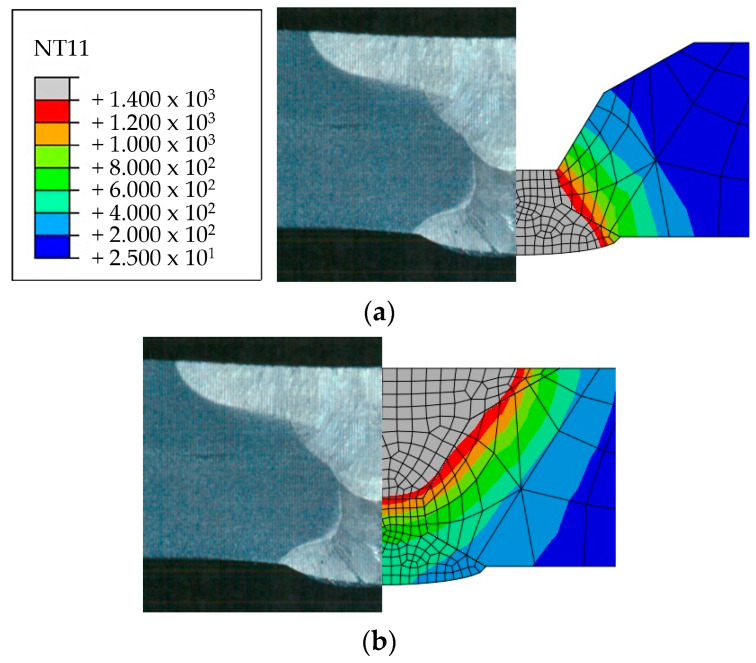
Comparison of experimental results and heat transfer analysis for Case 2: (**a**) 1 pass, (**b**) 2 pass.

**Figure 13:**
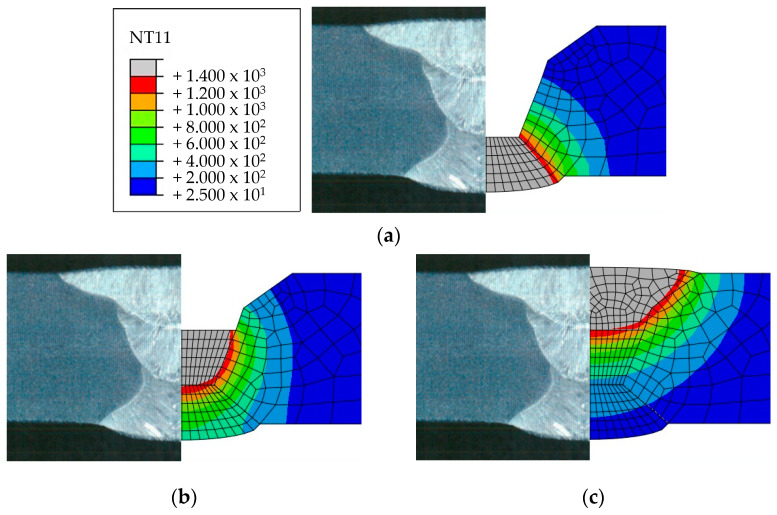
Comparison of experimental results and heat transfer analysis for Case 3: (**a**) 1 pass, (**b**) 2 pass, (**c**) 3 pass.

**Figure 14:**
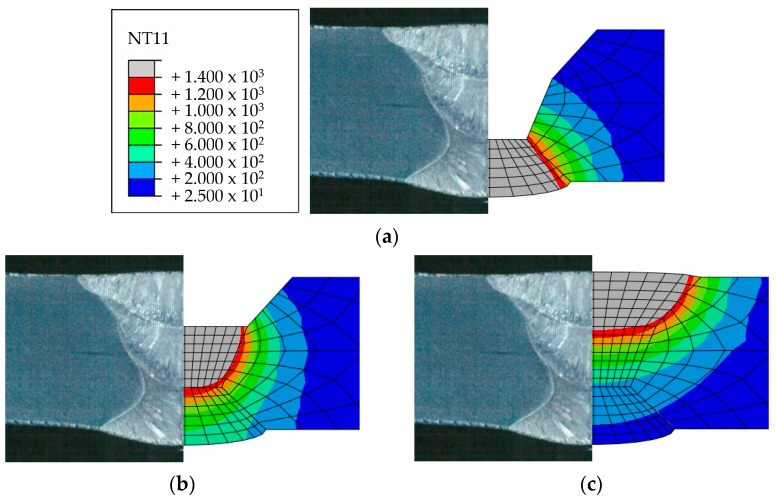
Comparison of experimental results and heat transfer analysis for Case 4: (**a**) 1 pass, (**b**) 2 pass, (**c**) 3 pass.

Heat transfer analysis was performed as described in Section 3.1 based on the welding conditions in Table 3. Figure 11, Figure 12, Figure 13 and Figure 14 show the temperature distribution when the heat source arrived for each case compared to Figure 7. The region above 1400 °C was assumed to be the welding zone [43,44,45]. By visual comparison, the depth of the fusion zone was similar, but there was a slight difference in its width.

To compare the analysis results with the experimental results in Section 2.2, the width and penetration length in each case were measured, as shown in Table 8. The values of 1 pass penetration and 1 pass width were consistent in all cases. The average error rate for penetration in all cases was 1.3%, and the highest error rate was 7.3% for 3 Pass penetration in Case 1. The average error rate for width was 10.5%, and the highest error rate was 13.9% for back bead width in Case 4. These results show that the method using UBHF and the block dumping technique effectively simulated the fusion melt zone according to the heat input size and demonstrated its effectiveness in identifying heat input trends within a short span of time.

**Figure 15:**
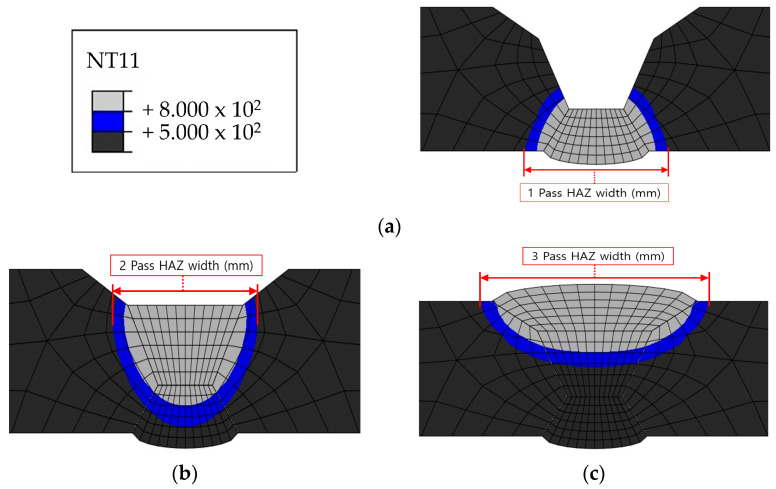
Heat-affected zone heat transfer analysis results for Case 1: (**a**) 1 pass, (**b**) 2 pass, (**c**) 3 pass.

**Figure 16:**
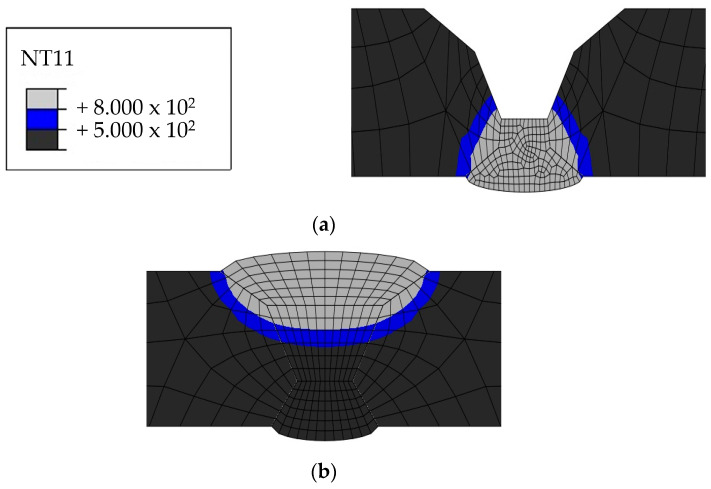
Heat-affected zone heat transfer analysis results for Case 2: (**a**) 1 pass, (**b**) 2 pass.

**Figure 17:**
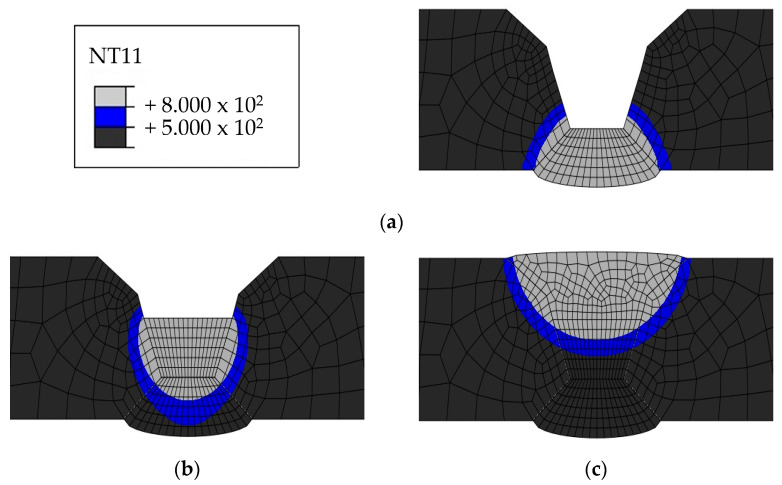
Heat-affected zone heat transfer analysis results for Case 3: (**a**) 1 pass, (**b**) 2 pass, (**c**) 3 pass.

**Figure 18:**
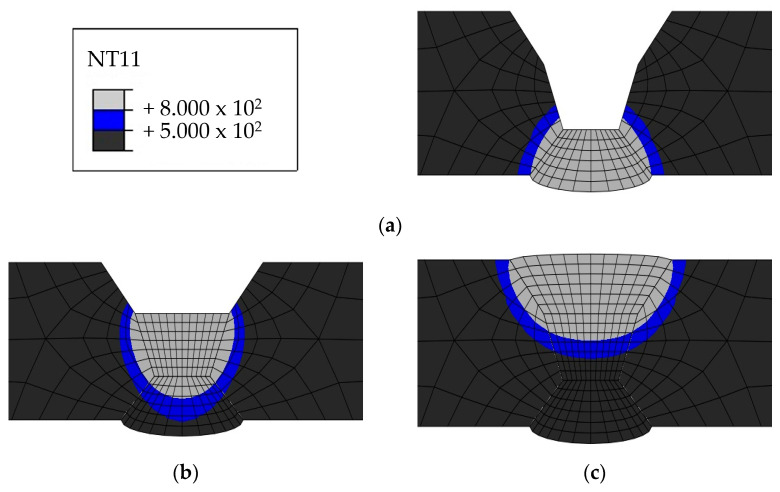
Heat-affected zone heat transfer analysis results for Case 4: (**a**) 1 pass, (**b**) 2 pass, (**c**) 3 pass.

The HAZ experiences temperatures ranging between 500 and 800 ℃ as it heats up and cools down. Therefore, the HAZ was examined because 316L is susceptible to intergranular corrosion after being exposed to a temperature range of 500–800 ℃. Figure 15, Figure 16, Figure 17 and Figure 18 show the size of the HAZ in each case, and Table 9 presents the measured lengths. Since the susceptibility to intergranular corrosion increases over time, the welding quality can be improved depending on the cooling method around the fusion zone.

## 5. Conclusions

This study performed 3 pass butt welding of 10 mm thick ASTM-A240M-316L using the FCAW process to investigate the effect of process variables through mechanical tests. Based on the experimental cross-sectional observation results, the size of the fusion zone and HAZ were examined using heat transfer FE analysis. The main findings are as follows.

- Tensile test results according to process variables showed that the yield strength was higher than the base metal in all cases. In Case 2, the tensile strength was 2.8% lower than the base metal at 1/3 position, but the average tensile strength was similar within 0.3%.
- The impact test results in a cryogenic environment (−196 ℃) were approximately 34.5–35.6 J for Cases 1, 3, and 4, and 44.7 J for Case 2. Although this disparity seems to be caused by the number of passes, repeated testing would be necessary to confirm the exact trend.
- Regarding welding deformation, Case 2 showed the least deformation, whereas Case 1 exhibited the most. The number of passes is believed to have the most significant influence on welding deformation. In addition, when comparing Cases 1, 3, and 4, the heat input of the first pass appeared to affect deformation more than the overall heat input.
- A moving heat source was simulated by combining the UBHF heat input model with the block dumping technique to reduce the time required for heat transfer FE analysis. In the fusion zone, the average error rate for penetration was 1.3%, while the average error rate for width was 10.5%. The HAZ was found to be about 20% larger than the fusion zone, and areas where intergranular corrosion could occur were identified. These results demonstrate that the method used in this study is effective for quickly assessing the temperature distribution of the heat input.

## Figures and Tables

**Table 1:** Chemical composition of ASTM-A240M-316L material.

Component	Percentage
Carbon, C (wt.%)	0.0176
Silicon, Si (wt.%)	0.590
Manganese, Mn (wt.%)	1.071
Phosphorus, P (wt.%)	0.0292
Sulfur, S (wt.%)	0.0050
Chromium, Cr (wt.%)	16.341
Nickel, Ni (wt.%)	10.111
Copper, Cu (wt.%)	0.278
Molybdenum, Mo (wt.%)	2.055
Nitrogen, N (ppm)	119

**Table 2:** Mechanical properties of ASTM-A240M-316L material.

Mechanical Properties	Value
Yield strength (MPa)	279
Tensile strength (MPa)	581
Elongation (%)	54

**Table 3:** Welding conditions of the FCAW experiment.

Case	Pass	Current (A)	Voltage (V)	Welding Speed (cm/min)
1	1	161	28	17.86
	2	165	29	29.13
	3 (Final)	143	28	24.79
2	1	167	31	15.23
	2 (Final)	224	24	15.96
3	1	155	28	19.23
	2	152	28	20.13
	3 (Final)	158	28	24.59
4	1	147	28	18.07
	2	149	29	18.52
	3 (Final)	154	29	18.63

**Table 4:** Cross-sectional observation and measurement results for each welding condition.

Case	Case 1	Case 2	Case 3	Case 4
Back bead height (mm)	0.928	0.928	1.060	1.031
Back bead width (mm)	8.925	10.486	10.795	10.898
1 pass penetration (mm)	2.975	3.428	2.580	2.769
1 pass width (mm)	4.624	4.595	4.507	4.963
2 pass penetration (mm)	4.831	6.572	3.654	3.991
2 pass width (mm)	9.764	17.923	7.482	8.409
3 pass penetration (mm)	2.194	-	3.770	3.240
3 pass width (mm)	17.511	-	15.272	14.433
Bead height (mm)	1.267	zero	0.398	0.353

**Table 5:** Results of tensile tests.

Case	Position	Yield Stress (MPa)	Tensile Stress (MPa)	Elongation (%)
Case 1	(1/3 Position)	348.9	609.8	34.3
	(2/3 Position)	348.7	606.4	36.0
	Average	348.8	608.1	35.2
	Base Material	300.1	591.1	58.8
Case 2	(1/3 Position)	328.1	577.4	35.1
	(2/3 Position)	343.0	606.9	38.9
	Average	335.6	592.2	37.0
	Base Material	304.8	593.6	59.1
Case 3	(1/3 Position)	340.6	611.0	37.0
	(2/3 Position)	345.8	608.5	34.1
	Average	343.2	609.8	35.6
	Base Material	305.6	592.9	60.9
Case 4	(1/3 Position)	344.9	610.7	36.1
	(2/3 Position)	333.4	609.0	36.1
	Average	339.2	609.9	36.1
	Base Material	301.1	591.9	59.8

**Table 6:** Results of impact test.

Case	Energy (J)			
	Position 1	Position 2	Position 3	Average
Case 1	36.1	32.9	34.6	34.5
Case 2	49.6	39.7	44.7	44.7
Case 3	39.4	33.3	34.1	35.6
Case 4	35.9	35.1	33.3	34.8

**Table 7:** Results of welding deformation measurement: (a) Case 1, (b) Case 2, (c) Case 3, (d) Case 4.

(a)						
Position	*X*-Axis (mm)					
*Y*-axis (mm)	10	40	80	120	160	200
0	1.760	3.600	7.722	11.664	15.692	19.844
100	4.020	5.900	9.672	13.598	17.476	21.512
200	5.560	7.362	10.984	14.890	18.660	22.746
300	5.270	6.962	10.694	14.430	18.300	22.174
400	3.870	5.550	9.222	13.056	16.824	20.688
500	1.240	2.830	6.700	10.704	14.330	18.350
**(b)**						
**Position**	***X*** **-axis (mm)**					
*Y*-axis (mm)	10	40	80	120	160	200
0	0.770	1.620	3.270	4.720	6.300	7.632
100	1.560	2.510	3.590	4.870	6.400	7.762
200	1.940	2.660	3.890	5.300	6.620	7.832
300	1.630	2.270	3.460	4.740	6.040	7.312
400	1.150	1.700	2.920	4.200	5.410	6.650
500	−0.200	0.450	1.610	3.180	4.450	5.560
**(c)**						
**Position**	***X*** **-axis (mm)**					
*Y*-axis (mm)	10	40	80	120	160	200
0	1.480	3.530	7.122	10.824	14.188	17.768
100	3.600	5.330	8.722	12.056	15.612	19.052
200	4.800	6.530	9.802	13.056	16.541	19.894
300	4.390	6.040	9.412	12.856	16.244	19.694
400	3.280	4.950	8.392	11.766	15.252	18.550
500	0.940	2.610	6.220	9.572	13.248	16.544
**(d)**						
**Position**	***X*** **-axis (mm)**					
*Y*-axis (mm)	10	40	80	120	160	200
0	0.420	1.740	4.530	7.102	9.662	12.396
100	2.340	3.580	6.030	8.512	10.984	13.578
200	3.500	4.660	6.992	9.512	11.896	14.380
300	2.960	4.180	6.570	8.822	11.294	13.808
400	1.730	2.770	5.170	7.542	9.894	12.446
500	0.640	0.400	2.740	5.240	7.692	10.194

**Table 8:** Measurement and comparison of dimensions: experimental results and heat transfer analysis results.

Case	Case 1		Case 2		Case 3		Case 4	
	Experiment	FEM	Experiment	FEM	Experiment	FEM	Experiment	FEM
Back bead width (mm)	8.925	8.295	10.486	8.499	10.795	9.262	10.898	9.567
1 pass penetration (mm)	2.975	2.975	3.428	3.428	2.580	2.580	2.769	2.769
1 pass width (mm)	4.624	4.624	4.595	4.595	4.507	4.507	4.963	4.963
2 pass penetration (mm)	4.831	4.831	6.572	6.463	3.654	3.715	3.991	4.020
2 pass width (mm)	9.764	8.906	17.923	14.046	7.482	6.972	8.409	7.735
3 pass penetration (mm)	2.194	2.366	-	-	3.770	3.770	3.240	3.486
3 pass width (mm)	17.511	15.623	-	-	15.272	12.621	14.433	13.079

**Table 9:** Measurement results of HAZ dimensions.

Case	Case 1	Case 2	Case 3	Case 4
1 pass HAZ width (mm)	10.859	12.573	12.576	13.035
2 pass HAZ width (mm)	11.698	19.806	9.569	10.445
3 pass HAZ width (mm)	19.648	-	16.900	16.927

## Data Availability

The data presented in this study are available on request from the corresponding author.
